# Tumour budding in invasive ductal breast carcinomas: correlation with clinicopathological prognostic parameters and hormone receptor status

**DOI:** 10.3389/pore.2025.1611983

**Published:** 2025-02-12

**Authors:** Sachin Sebastian Francis, Swati Sharma

**Affiliations:** Department of Pathology, Center of Basic Sciences, Kasturba Medical College, Manipal, Manipal Academy of Higher Education, Manipal, Karnataka, India

**Keywords:** invasive breast carcinoma, intra tumoral budding, peripheral tumour budding, prognostic parameters, Ki-67

## Abstract

**Introduction:**

Breast cancer is a leading cause of morbidity and mortality among women. Advances in molecular biology have improved detection and treatment, but conventional histopathological factors remain crucial for prognosis. Tumour budding, defined as clusters of less than 5 tumour cells detached from the main tumour, has been linked to poor prognosis in several cancers. This study explores the association between intra-tumoral budding (ITB) and peripheral tumour budding (PTB) with known prognostic factors in Invasive Breast Carcinoma of no special type (IBC NST).

**Materials and methods:**

This retrospective study analysed 70 cases of IBC NST diagnosed at Kasturba Medical College, Manipal, between January 2020 and December 2021. Tumour budding was classified as high-grade or low-grade based on density, which denotes the number of buds per x20 field. Clinicopathological data, including hormone receptor status, Ki-67 index, lymphovascular invasion (LVI), perineural invasion (PNI), and axillary lymph node involvement, were obtained. Statistical analyses were performed to identify a significant association between tumour budding and these factors. Univariate and multivariate logistic regression analyses were also done to demonstrate the significance of association.

**Results:**

High-grade PTB showed significant associations with LVI (p = 0.046), PNI (p = 0.017), and axillary lymph node involvement (p = 0.021). In contrast, high-grade ITB was only significantly correlated with axillary lymph node involvement (p = 0.044). LVI (p-value = 0.240) and axillary lymph node involvement (p-value = 0.142) did not show any association with PTB on multivariate analysis and PNI (p-value = 0.074) near significant association with PTB). A significant inverse association was observed between PTB and Ki-67 (p = 0.012), which remained significant in univariate and multivariate analysis (p-value = 0.017). No significant associations were found between tumour budding and hormone receptor status or menopausal status.

**Conclusion:**

Peripheral tumour budding (PTB) is significantly associated with several poor prognostic factors in IBC NST, while intra-tumoral budding (ITB) correlates primarily with axillary lymph node involvement. Tumor budding, particularly PTB, could serve as an important prognostic marker in breast cancer. Further research is needed to standardize tumour budding assessment in clinical practice.

## Introduction

Breast cancer is one of the most highly prevalent malignancies in women and causes considerable morbidity and mortality [1]. There were an estimated 20 million new breast cancer cases worldwide and 9.7 million cancer deaths in 2022, with the majority of them being in Asia [2]. The advances in genetics and molecular biology have improved the detection and treatment of breast carcinomas, which in turn have led to better outcomes. However, conventional histopathological prognostic parameters still play a crucial role in prognostication of breast cancers. Hence, identifying more meaningful and reliable histopathological factors to complement the current evaluation protocols is essential, and this is where tumour budding becomes relevant [3, 4].

Tumour budding is a pathological phenomenon associated with many cancers. Its definition varies from study to study but generally is defined as a cluster of 5 or fewer tumour cells which have detached from the bulk of the tumour and which don’t show features of differentiation [5]. They can be observed at the invasive margins of the tumour and are called peritumoral or peripheral tumour buds (PTB), while those that are seen in the tumour mass are called intra-tumoral buds (ITB) [6, 7].

At the molecular level, they are hypothesised to be the histological manifestations of epithelial-mesenchymal transition (EMT). EMT is thought to be a multi-step, dynamic phenomenon that occurs in epithelial cells, where they lose their ability to adhere to their neighbouring cell and develop migratory and invasive characteristics like mesenchymal cells [8]. Both EMT and its opposite process, mesenchymal-epithelial transition (MET), are physiological processes that are important for tissue repair, wound healing and embryonic development. The abnormal activation of EMT is now recognized as a key characteristic of cancer metastasis [8–10]. Hence, tumour budding was also thought to be a general invasive indicator and a poor prognostic factor.

The association between tumour budding with cancers were first described in 1949 by Imai with respect to gastric cancers [11]. Now, tumour budding is recognised as an aggressive and prognostic indicator in colorectal, oesophageal and gastric cancers [3, 12]. However, there is limited information about the role and relevance of tumour budding in breast cancers. So, this study attempts to correlate tumour budding, both intra-tumoral and peripheral tumour budding to known clinicopathological prognostic factors of breast carcinoma and hormone receptor status.

## Materials and methods

This retrospective observational study was done in the Department of Pathology, Kasturba Medical College, Manipal Academy of Higher Education, Manipal over 2 years from 1st January 2020 to 31st December 2021.

All cases diagnosed as Invasive Breast Carcinoma, no special type (IBC NST) and who underwent surgical resection (Radical or simple mastectomy) in our hospital were included. Patients who underwent core biopsy or lumpectomy, who received any pre-surgical therapy, who were identified to have distant metastasis at the time of primary tumour diagnosis or where clinical data and histopathological slides were unavailable, were excluded from the study. Patients with only core biopsy done were excluded from the study but the ones followed by radical or simple mastectomy were included in the study. Breast conservation surgery specimens were limited in number and were excluded.

The clinical details of these patients such as age, sex, presenting symptoms, details of previous therapy, radiological details and follow-up were retrieved from LIS/RISPACS/EMR discharge summaries and the Medical Records Department. The paraffin blocks and haematoxylin and eosin (H&E) stained slides for the corresponding case numbers of each patient were retrieved from the pathology archives. The gross features were analysed from the pathology reports in the database. The histopathological parameters, hormone receptor status and molecular subtype were analysed from archived pathology data and slides.

### Assessment of tumour budding

In this study, we defined tumour budding as an isolated single cancer cell or a cluster of up to 5 tumour cells detached from the main bulk of the tumour, showing no features of differentiation. In this study, we studied tumour budding

• At the invasive front (PTB)
• Inside the body of the tumour (ITB).

**FIGURE 1:**
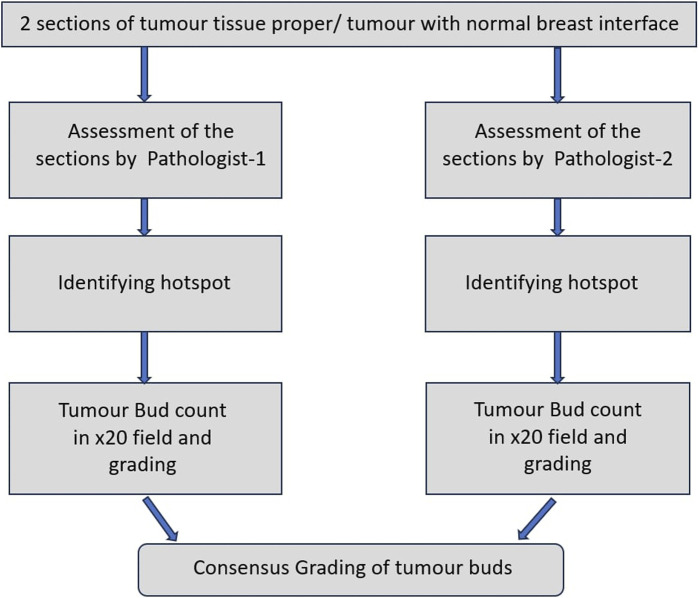
Work-flow diagram.

For all cases, 2 sections each of the tumour tissue proper and tumour with normal breast interface were studied for assessment of intra-tumoral and peripheral tumour budding respectively. This assessment was performed by both the pathologists, individually and later together to give a consensus value by studying the microscopy on a double-headed microscope. Hot spots were identified by studying the 2 sections each of the tumour proper and tumour with normal breast interface entirely. The intra- and peripheral tumour budding were counted on 20X field in the identified hot spot. This has been illustrated in Figure 1.

We separated the cases into 2 categories according to tumour bud density for both ITB and PTB per x20 field (tumour bud density) in hot spot areas [12].

• Low grade: <10 tumour buds per x20 field
• High grade: ≥10 tumour buds per x20 field

**FIGURE 2:**
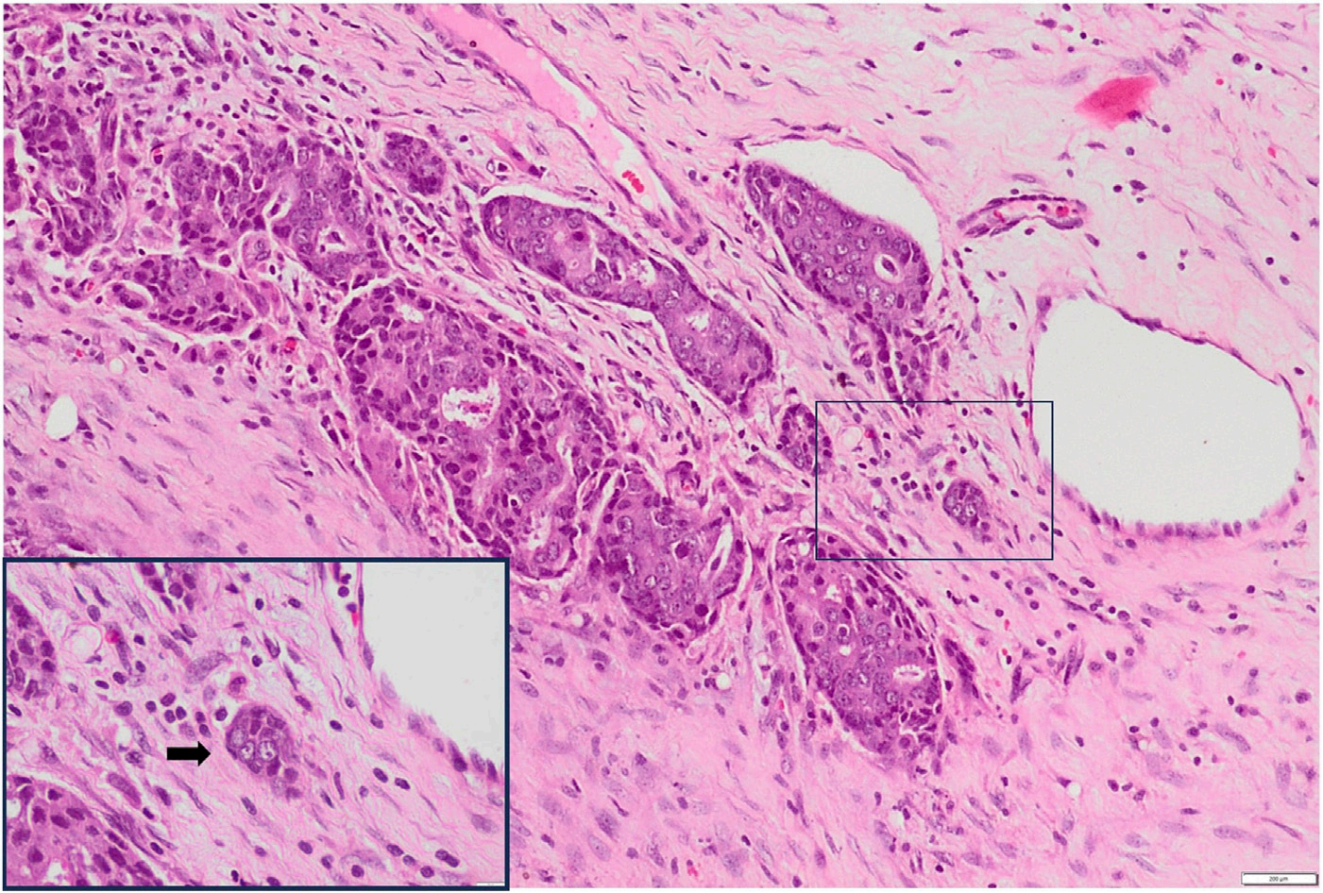
Low-grade PTB H&E X100 (Inset X400).

**FIGURE 3:**
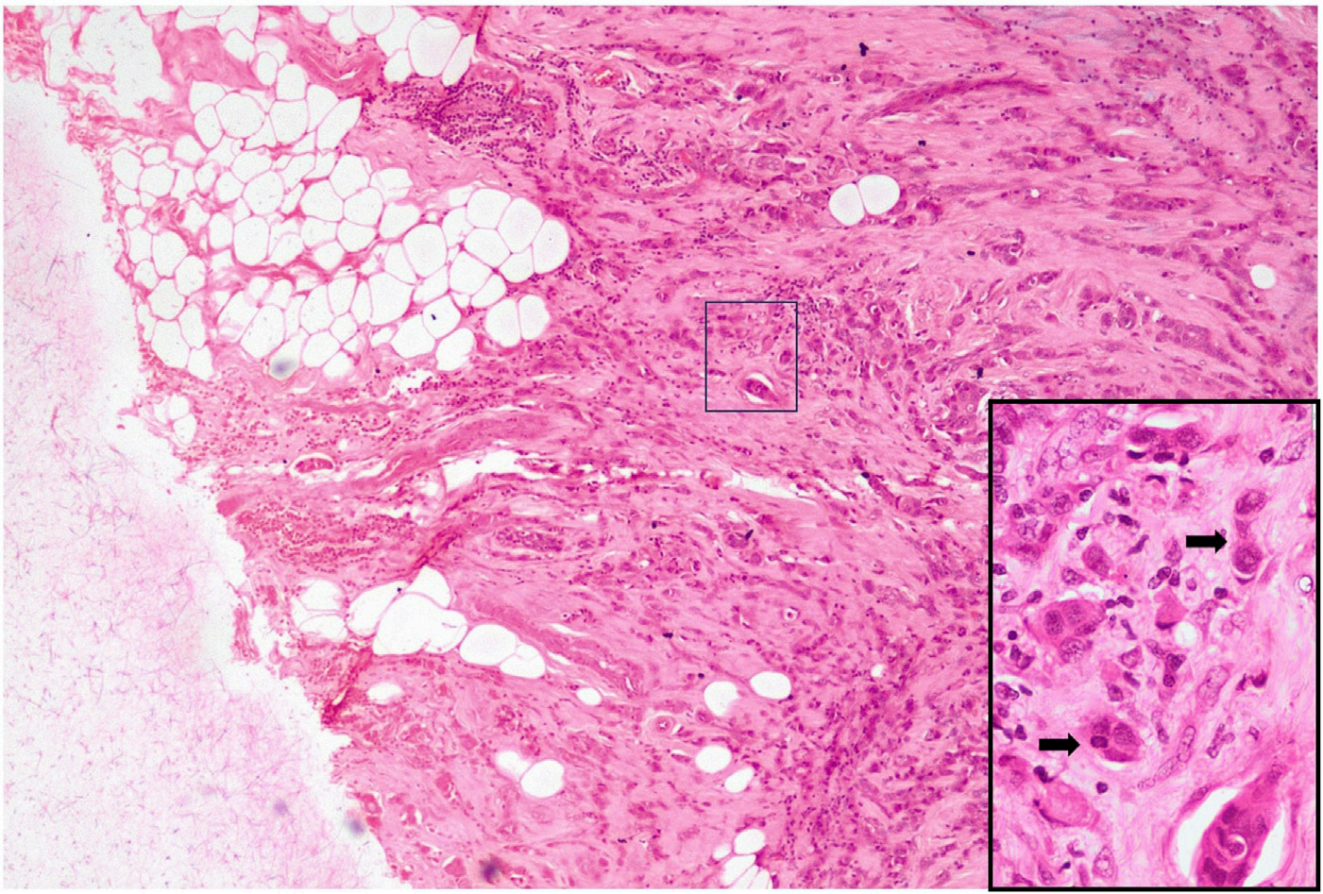
High-grade PTB H&E X40 (Inset X400).

**FIGURE 4:**
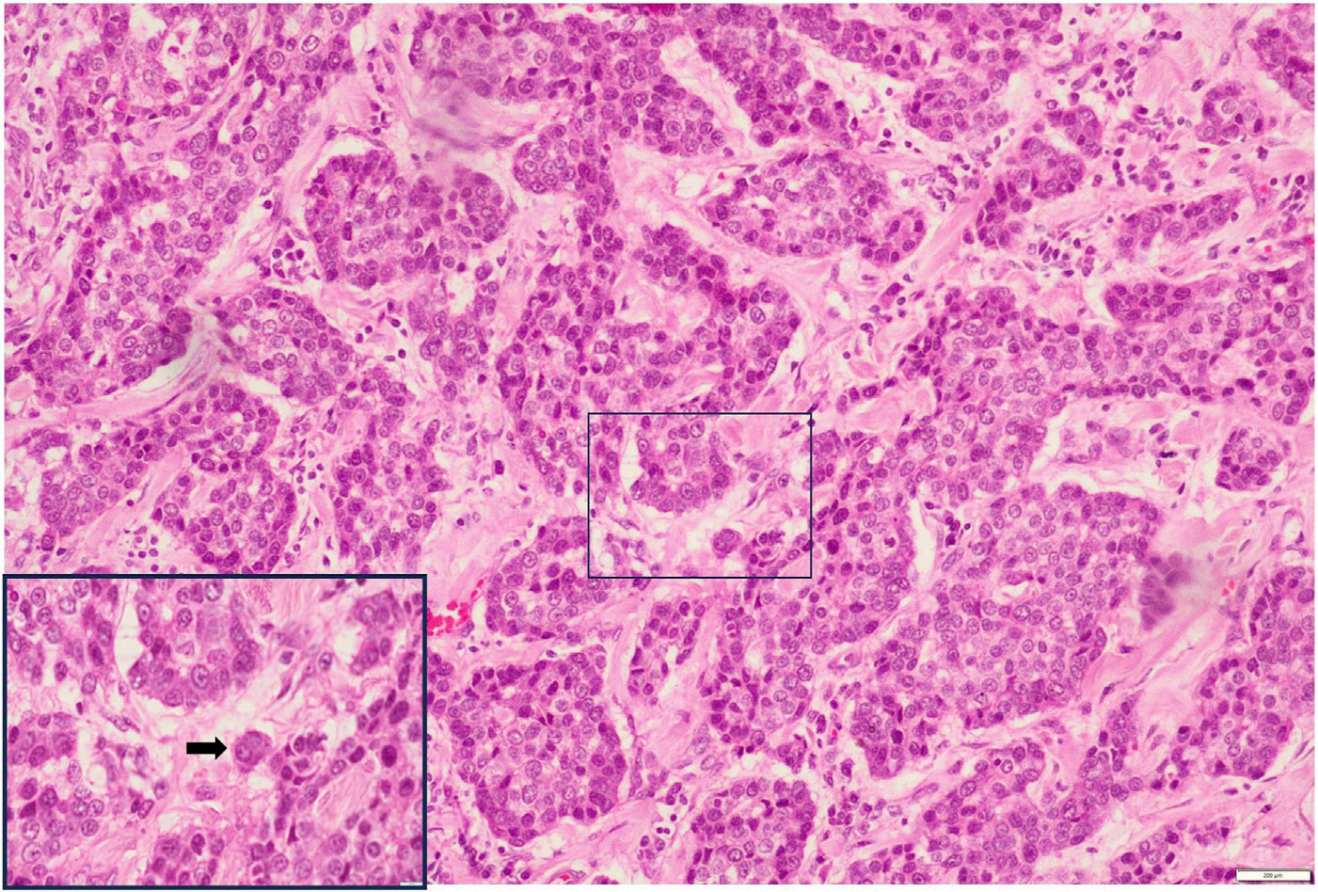
Low-grade ITB H&E X200 (Inset X400).

**FIGURE 5:**
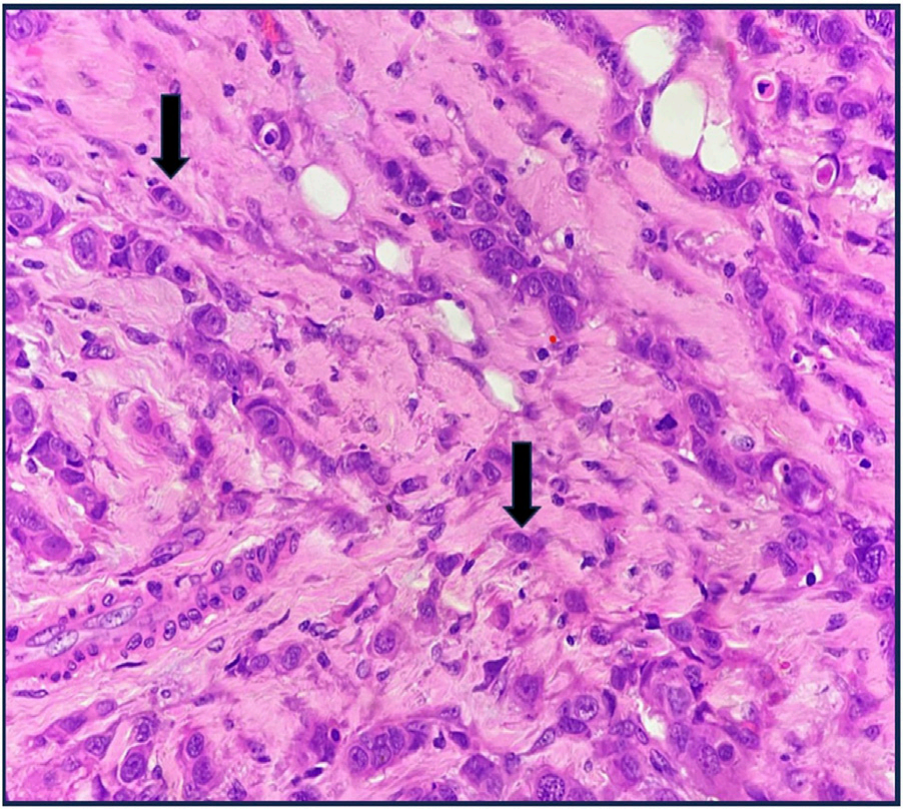
High-grade ITB H&E X400.

Slide images of low and high grade PTB and ITB are illustrated in Figures 2–5.

### Statistical analysis

The collected data were entered into Microsoft Excel 2016 and analysed using IBM SPSS Statistics for Windows, Version 27. The data descriptive statistics were described using frequency analysis, percentage analysis while categorical variables and the median & standard deviation were used for continuous variables. The significance of categorical data was ascertained using the Chi-Square test. The variables which were significant with the Chi-Square test were further analysed by univariate and multivariate logistic regression analyses. In all the above statistical tools a p-value of 0.05 or less is considered as significant level.

## Results

In this study, tumour budding in invasive breast carcinomas was studied for a period of 2 years with 70 cases. All the patients in this study were females and had a wide age distribution ranging from 25 to 85 years, with the mean age being 57.23 years. The majority of the patients, 29 (41.4%), were >60 years.

51.4% of the patients had attained menopause while 34 (48.6%) were pre or peri menopausal.

In the vast majority of the cases, 54 (77.2%) had the greatest tumour dimension of 2–5 cm.

Thirty-two cases (45.7%) were graded as Grade 2 according to the Nottingham Grading system, while 28 (40.0%) cases and 10 (14.3%) were graded as Grade 3 and Grade 1, respectively.

Out of 70 cases, only 29 (41.4%) cases were positive for lympho-vascular invasion as 10 (14.2%) cases showed perineural invasion. Five cases showed both LVI and PNI. Positive axillary lymph node metastasis was found in 41 (58.6%) cases.

ER and PR positivity was seen in 52 (74.3%) and 42 (60%) cases respectively. Her2neu was positive in 20 (28.16%) cases.

Ki-67 was categorised into 2 groups [13, 14]:

 • 0–20%- low
• >20%- high

The majority of the cases showed a high (>20%) Ki-67 index. Table 1 illustrates all the above clinicopathological parameters.

**TABLE 1:** Distribution of clinicopathological parameters.

Parameter	Frequency (n = 70)
Menstrual status: • Pre/Peri- menopausal • Post- menopausal	34 (48.6%) 36 (51.4%)
Tumour Size: • <2 cm • 2–5 cm • >5 cm	8 (11.4%) 54 (77.2%) 8 (11.4%)
Nottingham Grade: • Grade 1 • Grade 2 • Grade 3	10 (14.3%) 32 (45.7%) 28 (40.0%)
LVI	29 (41.4%)
PNI	10 (14.3%)
Axillary Lymph Node involvement: • Positive • Negative	41 (58.6%) 29 (41.4%)
Hormone Receptors: • ER+ • PR+ • HER2+	52 (74.3%) 42 (60%) 20 (28.16%)
Ki-67: • 0%–20% • >20%	12 (17.1%) 58 (82.9%)
Molecular Subtype: • Luminal A • Luminal B • HER2 enriched • TNBC	9 (12.9%) 46 (65.7%) 6 (8.5%) 9 (12.9%)
ITB: • Low • High	46 (65.7%) 24 (34.3%)
PTB: • Low • High	50 (71.4%) 20 (28.6%)

Both pre-menopausal and post-menopausal cases predominantly exhibited low-grade intra-tumoral budding (ITB) and peri-tumoral budding (PTB), with a slight increase in high-grade PTB and ITB observed in post-menopausal cases. However, the correlation between tumour budding and menopausal status was not statistically significant. Tumour budding generally remained low-grade across all tumour size groups, though high-grade ITB was present in 83.3% of tumours smaller than 2 cm. Despite this observation, p-values indicated no statistically significant difference in tumour budding distribution across different tumour sizes for ITB and PTB.

Grade 1 tumours showed a high proportion of low-grade ITB and PTB, while high-grade ITB was most prevalent in grade 2 tumours, and high-grade PTB was most common in grade 3 tumours. However, these correlations were not statistically significant. High-grade PTB was significantly associated with lymphovascular (p = 0.046) and perineural invasion (p = 0.017). In contrast, despite being more frequent in cases with LVI and PNI, high-grade ITB did not show a significant correlation. Both high-grade ITB (p = 0.044) and PTB (p = 0.021) were significantly linked to axillary lymph node involvement.

In terms of breast cancer subtypes, high-grade ITB and PTB were more frequently observed in Luminal A type cancers, with the lowest proportions found in triple-negative breast cancers. However, this correlation was not significant (p-values of 0.801 for ITB and 0.183 for PTB). High-grade tumour budding was more common in tumours with low Ki-67 index (0%–20%) for both ITB (41.7%) and PTB (58.3%), with the correlation being significant for PTB (p = 0.012).

LVI (p-value = 0.050), PNI (p-value = 0.025), axillary lymph node involvement (p-value = 0.027), and Ki-67 (p-value = 0.018), index showed significant association with PTB on univariate logistic regression analysis, while only Ki-67 (p-value = 0.017) index showed significant association with PTB on multivariate logistic regression analysis, although PNI (p-value = 0.074) showed near significance. Association of PTB with clinicopathological parameters, along with univariate and multivariate analysis are illustrated in Tables 2, 3.

**TABLE 2:** Correlation of PTB with clinicopathological parameters.

Parameter		PTB		p-value
		Low (n = 50)	High (n = 20)	
Menopausal status	Pre/Peri-Menopausal	25 (73.5%)	9 (26.5%)	0.705
	Post- Menopausal	25 (69.4%)	11 (30.6%)	
Tumour Size	<2 cm	4 (50%)	4 (50%)	0.361
	2–5 cm	40 (74%)	14 (26%)	
	>5 cm	6 (75%)	2 (25%)	
Nottingham Grade	Grade 1	8 (80%)	2 (20%)	0.764
	Grade 2	23 (71.9%)	9 (28.1%)	
	Grade 3	19 (67.9%)	9 (32.1%)	
LVI	Present	17 (58.6%)	12 (41.4%)	0.046
	Absent	33 (80.4%)	8 (19.6%)	
PNI	Present	4 (40%)	6 (60%)	0.017
	Absent	46 (76.6%)	14 (23.4%)	
Axillary LN involvement	Present	25 (61%)	16 (39%)	0.021
	Absent	25 (86.2%)	4 (13.8%)	
Molecular Subtypes	Luminal A	5 (55.5%)	4 (44.5%)	0.183
	Luminal B	30 (65.2%)	16 (34.7%)	
	HER2 Enriched	4 (66.6%)	2 (33.3%)	
	TNBC	7 (77.8%)	2 (22.2%)	
Ki-67 index	0%–20%	5 (41.7%)	7 (58.3%)	0.012
	>20%	45 (77.6%)	13 (22.4%)	

**TABLE 3:** Univariate and multivariate analysis of LVI, PNI, axillary lymph node involvement and Ki-67 with PTB.

Parameter	Univariate analysis		Multivariate analysis	
	Unadjusted OR with 95%CI	p-value	Adjusted OR with 95% CI	p-value
LVI	2.912 (1.00–8.48)	0.050	2.104 (0.608–7.277)	0.240
PNI	4.92 (1.21–19.97)	0.025	3.974 (0.874–18.058)	0.074
Axillary LN involvement	4.00 (1.17–13.65)	0.027	2.975 (0.694–12.750)	0.142
Ki-67 index	0.206 (0.056–0.759)	0.018	0.169 (0.039–0.725)	0.017

OR, Odds ratio.

CI, Confidence interval.

In case of ITB, only axillary lymph node involvement (p-value = 0.048) showed significant association on univariate analysis. Association of ITB with clinicopathological parameters and univariate analysis are illustrated in Tables 4, 5.

**TABLE 4:** Correlation of ITB with clinicopathological parameters.

Parameter		ITB		p-value
		Low (n = 46)	High (n = 24)	
Menopausal status	Pre/Peri-Menopausal	24 (70.5%)	10 (29.4%)	0.404
	Post- Menopausal	22 (61.1%)	14 (38.9%)	
Tumour Size	<2 cm	3 (16.7%)	5 (83.3%)	0.184
	2–5 cm	38 (70.3%)	16 (29.7%)	
	>5 cm	5 (62.5%)	3 (37.5%)	
Nottingham Grade	Grade 1	9 (90%)	1 (10%)	0.072
	Grade 2	17 (53.1%)	15 (46.9%)	
	Grade 3	20 (71.4%)	8 (28.6%)	
LVI	Present	16 (55.1%)	13 (44.9%)	0.118
	Absent	30 (73.1%)	11 (26.9%)	
PNI	Present	4 (40%)	6 (60%)	0.064
	Absent	42 (70%)	18 (30%)	
Axillary LN involvement	Present	23 (56%)	18 (44%)	0.044
	Absent	23 (79.3%)	6 (20.7%)	
Molecular Subtypes	Luminal A	5 (55.5%)	4 (44.5%)	0.801
	Luminal B	30 (65.2%)	16 (34.7%)	
	HER2 Enriched	4 (66.6%)	2 (33.3%)	
	TNBC	7 (77.8%)	2 (22.2%)	
Ki-67	0%–20%	7 (58.3%)	5 (41.7%)	0.554
	>20%	39 (67.2%)	19 (32.8%)	

**TABLE 5:** Univariate analysis of LVI, PNI, axillary lymph node involvement and Ki-67 with ITB.

Parameter	Univariate analysis	
	Unadjusted OR with 95% CI	p-value
LVI	2.216 (0.810–6.062)	0.121
PNI	3.500 (0.880–13.918)	0.075
Axillary LN involvement	3.000 (1.009–8.921)	0.048
Ki-67	0.682 (0.191–2.433)	0.555

OR, Odds ratio.

CI, Confidence interval.

## Discussion

Breast carcinoma is one of the most prevalent cancers worldwide, contributing significantly to mortality and morbidity. Consequently, extensive research has been conducted to identify prognostic factors associated with the disease, aiming to reduce its impact. This study attempts to correlate tumour budding, which has been established as a prognostic factor in other carcinomas, with known clinicopathological prognostic factors for breast carcinoma.

All patients in this study were female, consistent with the findings of Agarwal et al. [12], Liang et al. [3], and Salhia et al. [15]. However, Silva et al. [16] reported 2% of cases being male and 98% female. In this study, 36 (51.4%) patients were post-menopausal, aligning with the findings of González et al. [17] and Mozarowski et al. [4]. In contrast, Agarwal et al. [12] reported that 55% of patients were pre-menopausal, a difference likely due to the fact that only 14 (35%) of cases in Agarwal’s study involved patients over 50, while in the present study, 29 (41.4%) were over 60 years old.

The tumour size distribution in this study, with a maximum dimension of 2–5 cm, mirrors the findings of Chandana et al. [18], Agarwal et al. [12] and Singh et al. [19] grouped tumours into two categories (≤5 cm and >5 cm) and similarly found that most tumours were 5 cm or smaller. Gujam et al. [20], however, found that most tumours were ≤2 cm, a difference that may be attributed to geographical and economic factors, as the study was conducted in the UK, where greater patient awareness and better access to screening lead to earlier diagnosis.

In this study, most cases were of Nottingham Grade 2 (n = 32, 45.7%), consistent with the findings of Salhia et al. [15] and Chandana et al. [18]. Similarly, Liang et al. [3] and Singh et al. [19] reported approximately 70% of cases as Grade 2. However, studies by Agarwal et al. [12], Muda et al. [21], and Rathod et al. [22] reported a higher proportion of Grade 3 tumours, which could be due to differences in sample size, inter-observer variability in applying grading criteria, temporal variations and population differences.

Lymphovascular invasion (LVI) was found in 29 (41.4%) cases, consistent with the findings of Agarwal et al. [12], Liang et al. [3], Salhia et al. [15], Gupta et al. [23], and Öztürk et al. [24]. However, discordance was noted in the studies by Muda et al. [21], Singh et al. [19], and Kumarguru et al. [25]. Perineural invasion was observed in only a minority of cases, similar to the findings of Salhia et al. [15], Muda et al. [21], and Öztürk et al [24].

Axillary lymph node status is a critical prognostic indicator in breast carcinoma. In this study, 41 (58.6%) cases showed axillary lymph node positivity, consistent with the observations of Agarwal et al. [12], Singh et al. [19], Rathod et al. [22], and Kumarguru et al [25]. Discordance was noted in the studies by Gupta et al. [23] and Chandana et al. [18], which reported lower lymph node positivity, likely due to smaller sample sizes and lower LVI frequency, which is positively associated with lymph node metastasis. However, LVI (p-value = 0.240) and axillary lymph node involvement (p-value = 0.142) did not show any association with PTB on multivariate analysis and PNI (p-value = 0.074) near significant association with PTB. Only axillary lymph node involvement (p-value = 0.048) showed a significant association with ITB on univariate analysis.

The majority of cases in this study were of the Luminal B subtype, similar to Silva et al. [16] findings. However, González et al. [17] and Masilamani et al. [26] reported a higher proportion of Luminal A cases, which could be attributed to variations in sample size, geographical, and ethnic differences in the population studied.

In correlation with patient age, both intra-tumoral budding (ITB) and peri-tumoral budding (PTB) exhibited higher-grade budding in older age groups, which aligns with the findings of Liang et al. [3], Gujam et al. [20], and González et al. [17] However, most studies, including this one, did not find a statistically significant association between tumour budding and age.

In this study, we observed a decrease in the proportion of high-grade PTB as tumour size increased, although this finding was not statistically significant (p = 0.361). This is contrary to the majority of studies, including those by Liang et al. [3], Agarwal et al. [12], Kumaraguru et al. [25], Öztürk et al [24], Silva et al. [16], and Muda et al. [21], which found that high-grade tumour budding was more common in larger tumours, with statistically significant associations. For ITB, a similar pattern of decreasing high-grade budding with increasing tumour size was observed (p = 0.184), while Singh et al. [19] reported increased high-grade budding in both small and large tumours. The discrepancy in findings may be because many cases with tumours smaller than 2 cm had positive axillary lymph node involvement or lymphovascular invasion, which could act as confounding factors.

No significant association between histologic tumour grade and tumour budding was found in either ITB or PTB. In ITB, a higher proportion of high-grade budding was observed in Grade 2 tumours, consistent with the findings of Singh et al. [19] and Salhia et al. [15], although the association in this study was not statistically significant. The lack of significance may be attributed to the smaller sample size. In PTB, high-grade budding increased with tumour grade (p = 0.764), which is in line with the findings of Agarwal et al. [12] and Muda et al. [21], although Liang et al. [3] reported more high-grade budding in Grade 2 tumours. Muda et al. [21] also found a statistically significant association.

This study found a greater proportion of high-grade PTB in Luminal A subtypes, consistent with the findings of Gujam et al. [20] and Masilamani et al. [26], while Öztürk et al. [24] reported more high-grade PTB in Luminal B cases. For ITB, Salhia et al. [15] reported greater high-grade budding in the Luminal A subtype, which is consistent with the findings of this study.

This study also explored the relationship between Ki-67 and tumour budding (both ITB and PTB), revealing a significant inverse association between PTB and Ki-67 (p = 0.012), which persisted in both univariate and multivariate analyses. Ki-67 serves as a marker of tumour proliferative activity, while tumour budding reflects the tumour’s invasive and metastatic potential. Studies on p16(INK4a) and Ki-67 have demonstrated a cessation of proliferative activity at the invasive front of tumours, suggesting that invasion is not synonymous with proliferation. Our findings align with this concept, as the inverse relationship between PTB and Ki-67 indicates a phenotypic shift in tumour cells from a proliferative state to an invasive one [27–29]. Liang et al. [3] had previously validated that budded cells exhibit lower proliferative activity than tumour cells in other areas of the tumour, consistent with studies on tumour budding in colorectal carcinomas.

The strength of this study lies in its comprehensive analysis of both ITB and PTB, examining their relationships with various established prognostic factors—a focus rarely undertaken in previous research. Notably, we demonstrated that PTB is significantly associated with axillary lymph node positivity, LVI, PNI, and Ki-67, while ITB showed a correlation with axillary lymph node positivity. However, in multivariate analysis, only the association between PTB and Ki-67 remained significant.

The retrospective nature and the incorporation of only 70 cases of IBC NST are the limitations of this study as the small sample size limits the generalizability of the findings in this study and the retrospective nature limits the analysis to existing data, which might not have included all variables of interest. This in turn limits the ability to control the confounding factors and biases inherent in the data collection process. As the study was conducted in a single tertiary care centre, the external validity of the results can be in question as the findings might not apply to other populations. Our study mentions the lack of a standard method for quantifying tumour budding. This could have affected the consistency and reliability of the results. We also did not employ any immunohistochemical stains to assist in detecting tumour buds. For this study, we probably excluded some of the clinically relevant subgroups, e.g., post-neoadjuvant therapy cases or cases with breast conservative surgery. According to us, this study primarily focused on histopathological parameters and incorporated only limited follow-up data. Variables like long-term and disease-free survival were not analysed, which could be crucial for understanding the prognostic significance of tumour budding.

## Conclusion

This study evaluated tumour budding in terms of peripheral and intra-tumoral budding, categorising them into low-grade and high-grade. Peripheral tumour budding was significantly associated with several poor prognostic factors in invasive breast carcinoma of no special type, including LVI, PNI, and axillary lymph node involvement, however, no significant association was demonstrated in multivariate analysis. In contrast, intra-tumoral budding showed a significant association only with axillary lymph node involvement. Additionally, Ki-67 was seen to be inversely associated with peripheral tumour budding, even on multivariate analysis, suggesting that tumour proliferation and invasion are distinct processes. Given its potential prognostic value, tumour budding could be considered for inclusion in histopathological reporting protocols for breast carcinomas to complement conventional prognostic parameters. Further research is needed to establish a precise definition of tumour budding, including the number of cells forming a bud, the location of budding, standardized quantification methods, and the use of immunohistochemical stains to enhance detection.

## Data Availability

The original contributions presented in the study are included in the article, further inquiries can be directed to the corresponding author.
